# Editorial

**DOI:** 10.1120/jacmp.v1i3.2639

**Published:** 2000-06-01

**Authors:** Bhudatt R. Paliwal

The clinical medical physics community is extremely fortunate to be practicing a field of expertise that has an explosion of new ideas shaping their future. Granted a major factor driving this burst of development is the availability of fast and optimized computational and imaging capabilities, however, it is the clinical medical physicist who ultimately tests the new tools and implements them for the benefit of the patient. Over the past decades the clinical medical physicist faced difficulty in finding a suitable medium of expression to archive and communicate their work to his fellow colleagues and to the medical community at large. The establishment of the Journal of Applied Clinical Medical Physics (JACMP) by the Board of Chancellors of the American College of Medical Physics was an extremely important and visionary undertaking. The membership of the American College of Medical Physics by virtue of their support of the College are the true benefactors and beneficiaries of the journal.

I am extremely impressed by the exceptionally professional quality of the JACMP. It truly reflects the caliber and in depth effort of the Chief Editor, Peter Almond, Associate Editor, Mike Mills and their Advisory Editorial Board. We are all grateful for their vision and the years of work they have put in to the selection of outstanding publisher and equally impressive format for the Journal. JACMP goes a long way to give expression to the clinical medical physics community.

I have no doubt that under the Editorship of Dr. Peter Almond and his Editorial Board JACMP will continue to provide a high level of forum for the clinical medical physicists. It will be read all around the world and help our fellow colleagues in hopefully even the under developed countries as well.

